# Befragung zum Vorhandensein palliativmedizinischen Wissens sowie palliativmedizinischer Strukturen in deutschen Notaufnahmen

**DOI:** 10.1007/s00101-023-01356-3

**Published:** 2023-11-23

**Authors:** Lennert Böhm, Jacqueline Schwartz, Mark Michael, Eva Diehl-Wiesenecker, Michael Bernhard, Martin Neukirchen

**Affiliations:** 1 Zentrale Notaufnahme, Universitätsklinikum Düsseldorf, Heinrich-Heine-Universität, Moorenstraße 5, 40225 Düsseldorf, Deutschland; 2 Arbeitsgruppe „Ethik“ der Deutschen Gesellschaft für Interdisziplinäre Notfall- und Akutmedizin (DGINA e. V.), Berlin, Deutschland; 3 Interdisziplinäres Zentrum für Palliativmedizin, Universitätsklinikum Düsseldorf, Heinrich-Heine-Universität, Düsseldorf, Deutschland; 4 Zentrale Notaufnahme und Aufnahmestation, Charité – Universitätsmedizin Berlin, Campus Benjamin Franklin, Freie Universität und Humboldt Universität zu Berlin, Berlin, Deutschland; 5 Klinik für Anästhesiologie, Universitätsklinikum Düsseldorf, Heinrich-Heine-Universität, Düsseldorf, Deutschland

**Keywords:** Notfallmedizin, Ärzte, Fragebogen, Sterbebegleitung, Palliativkonsil, Emergency medicine, Physicians, Survey, Terminal care, Palliative care

## Abstract

Patienten mit lebenslimitierenden/palliativen Erkrankungen stellen eine Herausforderung für die Klinische Akut- und Notfallmedizin dar, denn trotz zunehmend ausgebauter Strukturen der spezialisierten ambulanten Palliativversorgung (SAPV) ist die Notaufnahme bei Symptomexazerbationen oder Überlastung der Angehörigen häufig erste Anlaufstelle. Da hier ebenso lebenslimitierende Erkrankungen erstdiagnostiziert und Therapieziele angepasst werden, scheint eine frühzeitige Anbindung von Notfallpatient:innen an palliativmedizinische Strukturen prognostisch günstig. Ziel der vorliegenden Untersuchung war die Erhebung einer Istanalyse zur Verfügbarkeit palliativmedizinischer Basiskenntnisse sowie palliativmedizinischer Strukturen in der Klinischen Akut- und Notfallmedizin. Hierzu wurde über notfallmedizinische Blogs eine Online-Befragung an in Notaufnahmen tätige Ärzt:innen adressiert. Insgesamt konnten 383 vollständig ausgefüllte Fragebogen ausgewertet werden. Es zeigte sich, dass die Befragten häufig palliative Krankheitssituationen erleben und behandeln. Ambulante und stationäre Ressourcen sind hingegen nicht flächendeckend verfügbar, und dort, wo z. B. Konsildienste verfügbar sind, herrscht Uneinigkeit darüber, wann sie in Anspruch genommen werden sollten. Strukturen für den unmittelbaren Sterbeprozess sind größtenteils vorhanden, allerdings fehlen häufig die Ressourcen Zeit und Personal. Es besteht ein Interesse an palliativmedizinischer Fort- und Weiterbildung. Fazit: Da Notaufnahmen die entscheidende Schnittstelle zwischen der ambulanten und stationären Versorgung sind, können hier durch einen interdisziplinären und ganzheitlichen Ansatz frühzeitig Weichen für die weitere Versorgung gestellt werden, von dem Patient:innen mit Palliativbedarf profitieren können.

**Zusatzmaterial online:**

Den vollständigen Fragebogen finden Sie der Online-Version dieses Artikels (10.1007/s00101-023-01356-3) beigefügt.

## Abstract (en)

Patients with life-limiting or palliative illnesses represent a challenge for emergency departments because, despite the growing availability of specialized outpatient palliative care resources at home, patients often present during symptom exacerbations or when family caregivers become overwhelmed. Also, as life-limiting illnesses are frequently first diagnosed there and treatment goals are adjusted, it appears advantageous to establish early connections between emergency patients with palliative needs and palliative care resources. The objective of this study was to conduct a survey evaluating the availability of fundamental palliative care knowledge and palliative care structures in clinical acute and emergency medicine. For this purpose, an online survey was distributed via emergency medicine blogs, targeting physicians working in emergency departments. In total, 383 fully completed questionnaires were analyzed. It was found that the respondents often encounter patients with palliative needs. However, both outpatient and inpatient palliative resources are not universally accessible, and where, for instance, consultation services are available, there is a lack of consensus regarding the appropriate timing for their utilization. Structures for end of life care are largely in place, although time and personnel are often insufficiently available. There is an expressed interest in further education and training in palliative care. In conclusion, as emergency departments serve as the interface between outpatient and inpatient care, an interdisciplinary and holistic approach can be employed to lay the groundwork for ongoing palliative care, benefiting patients with palliative needs.

## Hintergrund und Fragestellung

In deutschen Notaufnahmen werden jährlich rund 21 Mio. Patient:innen behandelt [21]. Patient:innen mit akuten oder chronisch-progredienten lebenslimitierenden Erkrankungen, die unter einer hohen Symptomlast leiden und damit einen palliativmedizinischen Bedarf haben [2] stellen mit bis zu 10 % einen relevanten Anteil dieser Behandlungen dar [14, 26, 33].

Der zunehmende Ausbau ambulanter palliativer Strukturen ermöglicht es immer mehr Patient:innen, ihr Lebensende zu Hause zu verbringen [3]. Trotzdem können akut auftretende Symptomkrisen (z. B. Schmerzexazerbationen, Atemnot, Blutungen) zu einer Rettungsdienstalarmierung führen. Patientenverfügungen, eine vorrausschauend Behandlungsplanung („advance care planning“) und/oder Notfallausweise können dann helfen, Entscheidungen im Sinne der Patient:innen zu treffen, und ungewünschte Behandlungen und Krankenhauseinweisungen vermeiden. Fehlen diese Entscheidungshilfen jedoch, liegen vor Ort nichtbeherrschbare Probleme (z. B. hohe Symptomlast, überforderte Angehörige) vor, oder ist das Rettungsdienstfachpersonal palliativmedizinisch unerfahren, so wird die Behandlung dann in ein Krankenhaus verlagert [7, 30–32]. Daher werden in Notaufnahmen tätige Ärzt:innen häufig mit palliativmedizinischen Fragestellungen konfrontiert. Abhängig von der Krankenhausstruktur können sie hierbei auf innerklinische Ressourcen wie Palliativstationen oder Palliativdienste zurückgreifen. Ohne diese, und ohne Übernahmemöglichkeit auf eine Normalstation, werden Symptomkontrolle und ggf. sogar die Finalphasenbegleitung in der Notaufnahme notwendig.

Durch *zeitgerechte Integration* der Palliativmedizin in die Behandlung von Patient:innen mit lebenslimitierender Erkrankung können frühzeitig Weichen in der Behandlung gestellt werden, die die Lebensqualität verbessern und die Lebenszeit verlängern [23]. Hierfür ist jedoch die adäquate Identifikation dieses Bedarfs, auch durch die Ärzt:innen der Notaufnahme, notwendig. Ziel der vorliegenden Untersuchung war es daher, eine aktuelle Istanalyse zu palliativem Wissen und palliativer Haltung der in Notaufnahmen arbeitenden Ärzt:innen, der ihnen verfügbaren palliativmedizinischen Ressourcen und des Umgangs mit sterbenden Patient:innen in deutschen Notaufnahmen mittels einer Online-Umfrage zu erheben.

## Material und Methode

### Fragebogendesign und Durchführung der Befragung

Es wurde ein Fragebogen entwickelt, der Fragen zu den folgenden Themenbereichen beinhaltete: (A) Biografie der Proband:innen, (B) palliatives Wissen, (C) palliative Haltung, (D) innerklinische palliativmedizinische Ressourcen sowie Trigger für ein Hinzuziehen des Palliativdienstes, (E) außerklinische palliativmedizinische Ressourcen und (F) Versorgung sterbender Patient:innen in der Notaufnahme (Zusatzmaterial online: ESM_Fragebogen Palliativmedizin in der Notaufnahme).

Die in einer früheren Arbeit unserer Arbeitsgruppe entwickelten Trigger für ein Hinzuziehen eines Palliativdienstes auf Intensivstationen [1] wurden für die Verwendung in der Notaufnahme abgewandelt. Zustimmung zu Statements konnte anhand einer 5‑stufigen Likert-Skala (5: stimme voll zu, 1: stimme gar nicht zu) erfolgen, biografische Angaben wurden gruppiert und mittels Multiple-Choice-Auswahl erfragt. Einzelne Fragen hatten die Möglichkeit einer Freitextangabe, falls keine der Antworten zutreffend war.

Aus dem finalisierten Fragebogen wurde eine Online-Befragung mittels des Umfrage-Tools LimeSurvey (Fa. LimeSurvey GmbH, Hamburg, https://www.limesurvey.org/de/) erstellt. Eine Einladung zur Teilnahme wurde am 02.11.2022 über die Geschäftsstelle der Deutschen Gesellschaft für Interdisziplinäre Notfall- und Akutmedizin e. V. (DGINA) an die im Mitgliederregister enthaltenen ärztlichen Mitglieder per E‑Mail versendet. Des Weiteren wurde auf den notfallmedizinischen Blogs www.news-papers.eu und www.nerdfallmedizin.de und über die Twitter-Kanäle aktiver Notfallmediziner:innen über die Befragung informiert und für eine Teilnahme geworben. Erinnerungen wurden 2 bis 3 Wochen nach der initialen Annoncierung erneut versendet. Eine Teilnahme war bis zum 31.12.2022 möglich.

Angesprochen wurden Ärzt:innen, die während des Befragungszeitraumes in einer Notaufnahme tätig waren; Ausschlusskriterium waren unvollständig ausgefüllte Datensätze der Online-Evaluation. Die Umfrage war freiwillig, erfolgte anonymisiert und enthielt keine finanziellen Incentives.

Ein positives Ethikvotum der Medizinischen Fakultät der Heinrich-Heine-Fakultät (No.: 2022-1951) lag vor.

### Datenaufbereitung und Auswertung

Die Befragungsdaten wurden zur Verarbeitung und zur Auswertung nach Excel (Fa. Microsoft, Redmond, WA, USA, Version 365) exportiert. Neben einer deskriptiven Auswertung der Antworten kamen Mittelwert ± Standardabweichung, Median und Minimal- und Maximalwerte zur Anwendung. Antworten auf offene Fragen wurden zunächst aufgelistet und anschließend in Antwortkategorien zusammengefasst.

## Ergebnisse

**Figure 1:**
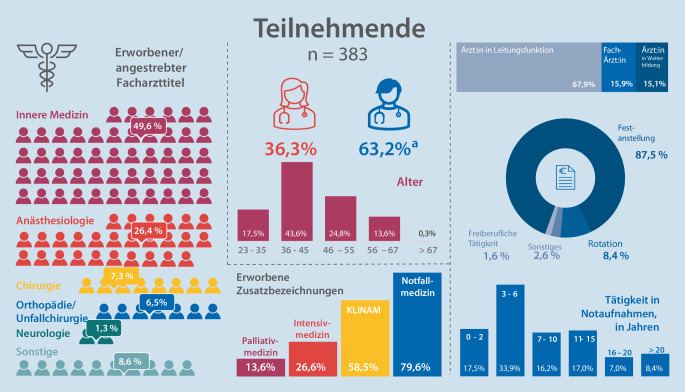

Über das Mitgliederregister der DGINA wurden 1425 E‑Mails verschickt. Die Reichweite der Aufrufe über Blogs und Twitter ließ sich nicht abschätzen. Insgesamt nahmen 640 Personen teil, *n* = 383 (59,8 %) der Fragebogen waren vollständig ausgefüllt und konnten ausgewertet werden. Die demografische Zusammensetzung, inkl. Alter, Geschlecht, Weiterbildung und Tätigkeitszeitraum in der Notaufnahme, wird in der Infografik zusammengefasst (Abb. 1).

### Erfahrung, Vorwissen und Haltung der Evaluationsteilnehmer:innen

Nur 121 (31,6 %) waren in Studium oder Weiterbildung über den Umgang mit sterbenden Patient:innen und ihren Angehörigen geschult worden; ein großer Anteil bildete sich aber auf eigene Initiative fort: Knapp über die Hälfte gaben an, im letzten Jahr einmal (115 [30,0 %]), 2‑ bis 3‑mal (*n* = 78 [20,4 %]) und mehr als 3‑mal (26, [6,8 %]) an palliativmedizinischen Fortbildungen zum Thema Palliativmedizin teilgenommen zu haben.

Patient:innen in palliativen Krankheitssituationen behandelten 83,5 % der Teilnehmer:innen „oft“. Dabei fühlten sich 289 (75,7 %) „eher sicher“ oder „sehr sicher“ und gaben an, „häufig“ Maßnahmen zur Symptomlinderung auszuführen.

**Figure 2:**
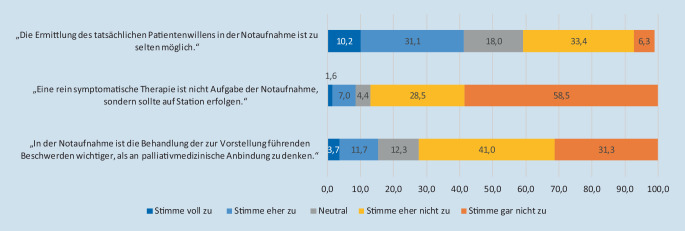

Die Mehrheit (333 [86,9 %]) sah eine symptomatische Therapie bei Palliativpatient:innen als Aufgabe der Notaufnahme, und ebenfalls eine Mehrheit (277 [72,3 %]) sah eine palliativmedizinische Anbindung von Palliativpatient:innen als wichtig an (Abb. 2).

### Verfügbarkeit palliativmedizinischer Dienste in der Notaufnahme

Die Möglichkeit, spezialisierten palliativmedizinischen Rat (z. B. in Form eines Palliativdienstes) einzuholen, gaben 265 (69,2 %) der Befragten an, und bei 61,9 % der Befragten werden auch Konsile in der Notaufnahme durchgeführt. Im Mittel wurden in diesen Notaufnahmen 34 ± 41 (Min-Max: 1–300) Patient:innen/Jahr dem Palliativdienst vorgestellt. Eine Mehrheit der Befragten ohne verfügbaren Palliativdienst (88 [74,6 %]) gab an, sich Unterstützung durch erfahrene Palliativmediziner:innen oder einen Palliativdienst in der Notaufnahme zu wünschen.

**Figure 3:**
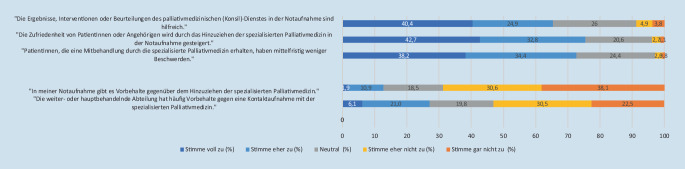

Die Einschätzungen zu positiven Effekten des Hinzuziehens von Palliativdiensten sowie auch zu potenziellen Vorbehalten finden sich in Abb. 3. Dass Patient:innen oder Angehörige in der Notaufnahme das Hinzuziehen eines Palliativdienstes ablehnen, kam bei 13 Befragten (4,9 %) „häufig“ und bei weiteren 55 (20,9 %) Befragten „gelegentlich“ vor.

### Trigger für ein Hinzuziehen des Palliativdienstes

**Figure 4:**
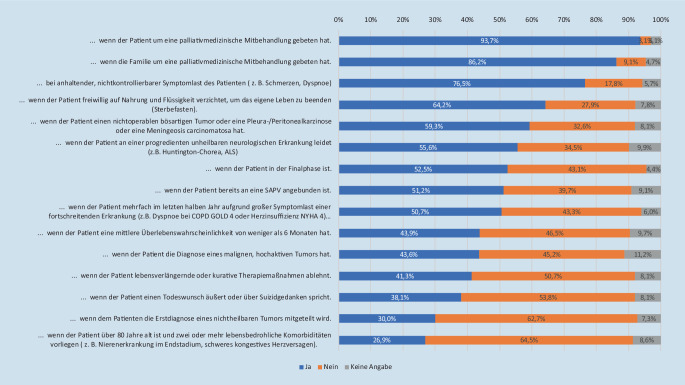

Die „surprise question“ („Wären Sie überrascht, wenn Ihr:e Patient:in innerhalb der nächsten 12 Monate versterben würde?“) [28] wird nur von einer Minderheit der Befragten (98 [25,6 %]) angewendet, um Patient:innen mit palliativem Bedarf zu identifizieren. Es wurden mehrere hypothetische Situationen abgefragt, ob diese zu einem Hinzuziehen des Palliativdienst führen würde, oder – wo dieser fehlte – ob Unterstützung durch erfahrene Palliativmediziner:innen gewünscht wäre. Hierbei fanden sich sehr unterschiedliche Zustimmungsraten (Abb. 4).

### Angebote der spezialisierten ambulanten Palliativversorgung

Fast alle Befragten (355 [92,7 %]) gaben an, dass in ihrer Stadt bzw. ihrem Kreis das Angebot einer spezialisierten ambulanten Palliativversorgung (SAPV) besteht. Aus der Notaufnahme heraus gaben 94 (26,5 %) an, eigene Patient:innen direkt in die Versorgung der SAPV anbinden zu können. Eine große Mehrheit (247, [69,6 %]) hatte dies aber „nicht“ oder „noch nicht“ versucht. Eine:n feste:n Ansprechpartner:in für das SAPV-Team zur Krankenhauseinweisung bei Versorgungsproblemen haben 144 (40,7 %) benannt. Nur 62 (16,2 %) gaben an, dass im Einzugsbereich ihrer Notaufnahme standardisierte Verfügungen für den Notfall (z. B. Palliativ- oder Notfallausweise) existieren, um den Rettungsdienst über bestehende Therapielimitierungen zu informieren.

### Sterben in der Notaufnahme

**Figure 5:**
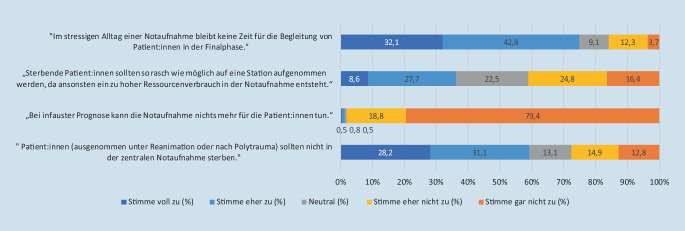

Ein Großteil (98,2 %) widersprach der Aussage, dass die Notaufnahmen bei infauster Prognose nichts mehr für Patient:innen tun können. Die weiteren Antworten zur Haltung der Befragten bezüglich Patient:innen, die in der Notaufnahme versterben, finden sich in Abb. 5**.**

Insgesamt 43 Befragte (11,2 %) gaben an, dass es in ihrer Notaufnahme die Möglichkeit gibt, eine Bezugspflegekraft für die Begleitung von Patient:innen in der Finalphase freizustellen. Die Räumlichkeiten, in denen diese Begleitung durchgeführt werden kann, unterschieden sich stark, und finden sich in Tab. 1. Keine geeigneten abgetrennten Räume gaben 46 der Befragten (12 %) an.

**Table 1:** 

Antwort		Anzahl	%
Ein eigenes Palliativzimmer für Patient:innen in der Finalphase und ihre Angehörigen		13	3,4
Es gibt einen Abschiedsraum, in dem sich trauernde Angehörige verabschieden können		44	11,5
Es gibt Einzelzimmer in der Notaufnahme, die genutzt werden können		263	68,7
Es stehen nur Untersuchungskabinen zur Verfügung		77	20,1
Freitextangaben	Notaufnahme‑/Beobachtungsstation	14	3,7
	Abschiedsraum in räumlicher Nähe	2	–
	Notfallbett der Palliativstation	1	–
	Funktionsraum der Notaufnahme (Gips‑, Schock‑, Isolationsraum)	4	–

Angaben zur Häufigkeit von Todesfällen in der zentralen Notaufnahme sowie zur Verlegung sterbender Patient:innen finden sich in Tab. 2**.**

**Table 2:** 

Häufigkeit	Anzahl	%
*Frage: Wie oft sterben Patient:innen (ausgenommen Patient:innen unter Reanimation oder Polytrauma) in ihrer Notaufnahme?*		
Mehr als 1/Woche	26	6,8
Wöchentlich	29	7,6
Ein- bis 3‑mal/Monat	121	31,6
Weniger als 1/Monat	187	48,8
Nie	7	1,8
Weiß nicht/keine Angabe	13	3,4
*Frage: Patient:innen in der Finalphase werden in meiner Notaufnahme auf Station verlegt*		
Immer	50	13,1
Häufig	200	52,2
Gelegentlich	79	20,6
Selten	44	11,5
Nie	4	1,0
Weiß nicht/keine Angabe	6	1,6

Die Möglichkeit, nach belastenden Situationen ein Kriseninterventionsteam oder eine/n Notfallseelsorger:in hinzuzuziehen, hatten 300 Befragte (78,3 %). Eine regelmäßige Supervision für Kolleg:innen nach belastenden Situationen gaben 81 (21,1 %) der Befragten an. Das Angebot eines Trainings für die Gesprächsführung in schwierigen medizinischen Situationen hatten nur 44 (11,5 %) absolviert.

## Diskussion

Die vorliegende Online-Evaluation stellt erstmals eine Istanalyse zu palliativem Wissen und palliativer Haltung in Notaufnahmen arbeitender Ärzt:innen, verfügbaren palliativmedizinischen Ressourcen und dem Umgang mit sterbenden Patient:innen in deutschen Notaufnahmen dar.

Besonders interessant ist die hohe selbst wahrgenommene Sicherheit der 383 Befragten bei der Behandlung von Patient:innen in Palliativsituationen. Sicherlich ist dies zu einem gewissen Teil auf einen *Selection Bias* bei der Teilnahme an einer Online-Befragung zu palliativmedizinischen Themen zurückzuführen. Dass ein relevanter Anteil der sehr erfahrenen Teilnehmenden mit einem hohen Anteil in Führungspositionen angab, Palliativmedizin nicht im Studium gelehrt worden zu sein, liegt sicherlich daran, dass diese erst seit 2013 als obligates Fach im Medizinstudium gelehrt und geprüft wird. Der ebenfalls geringe Anteil, der im Rahmen der ärztlichen Weiterbildung diesbezüglich geschult worden war, weist auf die bisher fehlende strukturierte Fortbildung hin, sodass die erwähnte Sicherheit vermutlich eher aus der eigenen Erfahrung und einer individuellen Auseinandersetzung mit der Thematik resultiert.

Die geringe Nutzung der *Surprise question* („Wären Sie überrascht, wenn Ihr Patient innerhalb der nächsten 12 Monate versterben würde?“), legt nahe, dass es zahlreiche Notaufnahmepatient:innen gibt, deren Palliativbedarf unentdeckt bleibt [28]. Bei der Frage nach konkreten Situationen, in denen eine palliativmedizinische Vorstellung erfolgen würde, gab es große Unterschiede, ähnlich einer vergleichbaren Befragung unter Intensivmediziner:innen [1]: Eine hohe Zustimmung bestand bei einer früheren Palliativanbindung, wenn diese durch Patient:in und Angehörige gewünscht wurde, sowie bei hoher Symptomlast. Eine besonders hohe Ablehnung fand sich bei multimorbiden Patient:innen – obwohl diese am meisten unter einer Übertherapie in den letzten 30 Lebenstagen leiden [5, 8] – und auch gegenüber der Anbindung direkt bei Erstdiagnose eines nichtheilbaren Tumors, vielleicht aus Sorge, dass dies als „Zu-früh-Aufgeben“ wahrgenommen wird. Hier besteht offensichtlicher Aufklärungsbedarf, denn die zeitgerechte Integration der Palliativmedizin sowohl bei fortgeschrittener lebenslimitierender Erkrankung wie auch bei prognostiziertem langen Überleben, aber erwarteter oder bestehender hoher Symptomlast führt zu erhöhter Lebensqualität, ohne die Lebenszeit zu verkürzen [11, 22, 24], und beugt einer Übertherapie am Lebensende vor [18]. Aus diesem Grund sollten Ärzt:innen in Notaufnahmen im Erkennen von palliativmedizinischem Bedarf geschult werden, auch da im weiteren innerklinischen Verlauf deutlich seltener palliativmedizinische Konsultationen initiiert werden [13]. Standardisierte Screening-Tools, deren Anwendung in der Notaufnahme sich in einzelnen Untersuchungen als machbar erwiesen haben [10, 20, 25] können hier helfen.

Nur ein Viertel der Befragten konnte Patient:innen direkt aus der Notaufnahme heraus an die ambulante Struktur einer SAPV anbinden, obwohl dies eine frühe Rückkehr ins häusliche Umfeld ermöglichen würde, und die Ressourcen von Krankenhaus und Rettungsdienst durch seltenere zukünftige Inanspruchnahmen mit daraus resultierenden unnötigen und belastenden Krankenhauseinweisungen [15] schonen würde. Ebenfalls verfügte weniger als die Hälfte der Befragten über einen direkten Kontakt, falls das SAPV-Team bei Versorgungsproblemen eine stationäre Einweisung indiziert sah, obwohl dies transsektorelle Schnittstellenprobleme und Übergabeverluste vermeiden könnte.

Auch in der vorliegenden Untersuchung zeigt sich, dass in Akutsituationen der mutmaßliche oder tatsächliche Patientenwille häufig nur schwierig eruiert werden kann. Patientenverfügungen sind in der Notaufnahme und in Akutsituationen häufig entweder nicht verfügbar oder nicht anwendbar [6, 27]. Notfallausweise, die einen schnellen Überblick über gewünschte oder gerade nichtgewünschte Therapien geben könnten, liegen bisher flächendeckend noch nicht vor. Hier könnten künftige Projekte zur bundesweiten Vereinheitlichung und Verfügbarmachung helfen.

Der (sicherlich plakativen) Aussage, dass die Notaufnahme bei infauster Prognose nichts mehr tun könne, widersprachen die Befragten nahezu einstimmig. Allerdings herrschen große Unterschiede bei den verfügbaren örtlichen und personellen Ressourcen: Über eigene Räume (z. B. Palliativzimmer, Abschiedsräume) oder die personelle Ressource, eine Bezugspflegekraft für die alleinige Finalphasenbegleitung freizustellen, verfügten nur wenige. Hier besteht deutlicher Verbesserungsbedarf, und während dieser in Neubauten umgesetzt werden könnte, könnten bis dahin möglicherweise auch durch flexibel einsetzbare Palliativsets (z. B. farblich angepasster Paravent zur Abtrennung und dimmbare indirekte Lichtquellen) würdigere Bedingungen geschaffen werden. Während es eine europäische Leitlinie mit klaren Empfehlungen für die Versorgung dieser Patient:innen gibt [9] und kürzlich auch ein Konsensuspapier zu palliativmedizinischen Aspekten in der Akut- und Notfallmedizin [16] veröffentlicht wurde, fehlt eine nationale Leitlinie zur Versorgung von Patient:innen am Lebensende in Notaufnahmen bisher.

Eine strukturierte Nachbereitung oder Supervision war in den wenigsten Notaufnahmen vorhanden, obwohl der Umgang mit schwerstkranken und sterbenden Patient:innen, inkl. Entscheidungen zur Therapiezieländerung, zu Erschöpfung, Beziehungsmüdigkeit, persönlicher Unzufriedenheit und im schlimmsten Fall zum Burn-out führen kann [12] und besonders im hektischen Umfeld einer Notaufnahme ohne Pausen und Möglichkeit zum Innehalten droht [17, 19]. Hier ist ein strukturiertes Umdenken erforderlich, da ärztliches Wohlbefinden das Wohlbefinden der Patient:innen bedingt [4, 29]. Sowohl das Notaufnahmepersonal als auch die ihnen anvertrauten Patient:innen würden daher von etablierten regelmäßigen ethischen Fallbesprechungen und Supervisionen profitieren.

Limitationen der vorliegenden Online-Befragung sind die fehlende Information darüber, wie viele Personen durch den initialen Aufruf erreicht wurden, sodass keine Rückschlüsse auf die Rücklaufquote möglich sind, sowie die fehlende Validiertheit des Fragebogens. Durch die Freiwilligkeit könnte ggf. ein Selection Bias aufgrund besonders motivierter und palliativmedizinisch orientierter Ärzt:innen vorliegen. Aufgrund des identifizierten Bedarfs an palliativmedizinischer Fort- und Weiterbildung ergibt sich die Notwendigkeit der Reevaluation und erneuten Überprüfung in einem gegebenen Zeitintervall. Ebenfalls wäre eine vergleichbare Untersuchung unter dem Pflegepersonal in Notaufnahmen interessant, um auch hier die Haltung und evtl. bestehenden Schulungsbedarf erfassen zu können.

## Fazit für die Praxis

- Palliativmedizinische Aspekte haben eine hohe Relevanz für die Klinische Akut- und Notfallmedizin, da häufig palliativmedizinische Patient:innen in Notaufnahmen versorgt werden.
- Standardisierte Screeningtools sollten angewendet werden, um Patient:innen mit palliativmedizinischem Bedarf identifizieren.
- Es besteht Bedarf an strukturierten Schulungs- und Fortbildungsangeboten.
- Gute transsektorelle Kommunikation verbessert die Versorgung von Palliativpatient:innen.
- Zukünftig sollten Neubauten von Notaufnahmen angemessene räumliche Ressourcen zur Versorgung sterbender Patient:innen wie z. B. Abschiedszimmer beinhalten. Bis zu deren Verfügbarkeit können flexibel einsetzbare Palliativsets Abhilfe schaffen.
- Flächendeckend ist die Einführung eines bundeseinheitlichen Notfallausweises zu fordern.
- Eine strukturierte, ethische Nachbereitung herausfordernder Fälle mit dem gesamten Notfallteam sollte in Notaufnahmen als regelmäßiger Termin etabliert werden.

### Supplementary Information




