# Supplementary material for: Survey on the presence of palliative care knowledge and palliative care structures in German emergency departments
Source: Anaesthesiologie. 2023 Nov 23;72(12):863–70. [Article in German] doi: 10.1007/s00101-023-01356-3 (PMC10692016; doi:10.1007/s00101-023-01356-3)
Supplement: Supplementary file 1 [file 101_2023_1356_MOESM1_ESM.pdf]

Zusatzmaterial zum Beitrag „Befragung zum Vorhandensein palliativmedizinischen Wissens sowie palliativmedizinischer Strukturen in deutschen Notaufnahmen“ von Böhm L, Schwartz J, Michael M et al. (2023) in *Die Anaesthesiologie.*

Beitrag und Zusatzmaterial stehen Ihnen auf www.springermedizin.de zur Verfügung. Bitte geben Sie dort den Beitragstitel in die Suche ein.

Befragung zum IST-Zustand palliativmedizinischen Wissens und zur Verfügbarkeit palliativmedizinischer Ressourcen in deutschen Notaufnahmen

Biographische Angaben

1. **Wie alt sind Sie?**
   a) 23 - 35 Jahre
   b) 36 - 45 Jahre
   c) 46 – 55 Jahre
   d) 56 – 67 Jahre
   e) 67 Jahre und älter

2. **Welches Geschlecht haben Sie?**
   □ männlich □ weiblich □ divers

3. **Welchen Facharzt haben Sie erworben bzw. streben Sie an?**
   a) Innere Medizin
   b) Chirurgie
   c) Orthopädie/Unfallchirurgie
   d) Anästhesiologie
   e) Neurologie
   f) Sonstiger: ________________

4. **Wie würden Sie Ihre Anstellung in der Notaufnahme beschreiben?**
   a) Festanstellung
   b) Freiberufliche Tätigkeit
   c) Gestellung aus anderer Klinik des Hauses in Rotation
   d) Anders, nämlich: ______________

5. **Wie viele Jahre sind Sie bereits in Notaufnahmen tätig?**
   a) 0 – 2 Jahre
   b) 3 - 6 Jahre
   c) 7 – 10 Jahre

d) 11 – 15 Jahre
e) 16 – 20 Jahre
f) Mehr als 20 Jahre

6. **Sie sind tätig als:**
   a) Arzt/Ärztin in Weiterbildung
   b) Facharzt/Fachärztin
   c) Arzt/Ärztin in Leitungsfunktion

7. **Welche der folgenden Zusatzbezeichnungen besitzen Sie (Mehrfachnennung möglich):**
   a) Palliativmedizin
   b) Klinische Akut- und Notfallmedizin
   c) Intensivmedizin
   d) Notfallmedizin

8. **Wie oft haben Sie im letzten Jahr an Fortbildungen (inkl. einzelnen Vorträgen im Rahmen z.B. größerer Kongresse) mit palliativmedizinischem Inhalt teilgenommen?**
   a) Gar nicht
   b) einmal
   c) 2-3 mal
   d) häufiger

Palliatives Wissen und Kompetenz

Bitte schätzen Sie Ihre Einstellung zu den folgenden Fragen / Feststellungen ein:

9. „Ich behandele in der Notaufnahme oft Patienten mit einer palliativen Krankheitssituation.“

a) Stimme voll zu
b) Stimme eher zu
c) Neutral
d) Stimme eher nicht zu
e) Stimme gar nicht zu

10. „Ich fühle mich im Umgang mit Palliativpatienten sicher.“

a) Stimme voll zu
b) Stimme eher zu
c) Neutral
d) Stimme eher nicht zu
e) Stimme gar nicht zu

11. „Ich führe an solchen Patienten häufig palliative Maßnahmen zur Symptomkontrolle durch (z. B. Morphingabe bei Dyspnoe).“

a) Stimme voll zu
b) Stimme eher zu
c) Neutral
d) Stimme eher nicht zu
e) Stimme gar nicht zu

12. „Ich wurde in meinem Studium oder meiner Weiterbildung auf die Behandlung und den Umgang mit sterbenden Patienten und deren Angehörigen geschult.“

a) Stimme voll zu
b) Stimme eher zu
c) Neutral
d) Stimme eher nicht zu
e) Stimme gar nicht zu

13. „Ich wende in meiner täglichen Praxis die „surprise question“ („Wären Sie überrascht, wenn Ihr Patient innerhalb der nächsten (6)12 Monate versterben würde?“) an, um potentielle Palliativpatienten zu identifizieren“

a) Stimme voll zu
b) Stimme eher zu
c) Neutral
d) Stimme eher nicht zu
e) Stimme gar nicht zu

Palliative Haltung

Bitte schätzen Sie Ihre Einstellung zu den folgenden Fragen / Feststellungen ein:

14. „Eine rein symptomatische Therapie ist nicht Aufgabe der Notaufnahme, sondern sollte auf Station erfolgen."

a) Stimme voll zu
b) Stimme eher zu
c) Neutral
d) Stimme eher nicht zu
e) Stimme gar nicht zu

15. „In der Notaufnahme ist die Behandlung der zur Vorstellung führenden Beschwerden wichtiger, als an palliativmedizinische Anbindung zu denken."

a) Stimme voll zu
b) Stimme eher zu
c) Neutral
d) Stimme eher nicht zu
e) Stimme gar nicht zu

16. „Bei infauster Prognose kann die Notaufnahme nichts mehr für den Patienten tun."

a) Stimme voll zu
b) Stimme eher zu
c) Neutral
d) Stimme eher nicht zu
e) Stimme gar nicht zu

17. „Sterbende Patienten sollten so rasch wie möglich auf eine Station aufgenommen werden, da ansonsten ein zu hoher Ressourcenverbrauch in der Notaufnahme entsteht."

a) Stimme voll zu
b) Stimme eher zu
c) Neutral
d) Stimme eher nicht zu
e) Stimme gar nicht zu

Innerklinische Ressourcen

Im Folgenden wollen wir Ihnen einige Fragen zur Verfügbarkeit palliativmedizinischer Expertise in Ihrer Klinik stellen.

18. Gibt es in Ihrem Krankenhaus die Möglichkeit, spezialisierten, palliativmedizinischen Rat durch Kollegen mit der Zusatzweiterbildung „Palliativmedizin“ einzuholen (z.B. im Rahmen eines palliativmedizinischen (Konsiliar)-Dienstes?

a) Ja
b) Nein

Falls Sie ja angegeben haben, beantworten Sie bitte die folgenden Fragen:

19. Der Palliativmedizinische Dienst kommt in die Notaufnahme, wenn er um ein Konsil gebeten wird.

a) Ja
b) Nein
c) Keine Angabe/Weiß nicht

20. Wie viele Konsile werden im Jahr durch die Notaufnahme an den Palliativmedizinischen (Konsil)-Dienst gestellt?

rund ________________

21. Die Ergebnisse, Interventionen oder Beurteilungen des palliativmedizinischen (Konsil)-Dienstes in der Notaufnahme sind hilfreich.

a) Stimme voll zu
b) Stimme eher zu
c) Neutral
d) Stimme eher nicht zu
e) Stimme gar nicht zu

22. In meiner Notaufnahme gibt es Vorbehalte gegenüber dem Hinzuziehen der spezialisierten Palliativmedizin.

a) Stimme voll zu
b) Stimme eher zu
c) Neutral
d) Stimme eher nicht zu
e) Stimme gar nicht zu

Bei Zugriff auf einen palliativmedizinischen Konsiliardienst (Frage 18 mit „ja“ beantwortet): Auslösende Faktoren für Mitbehandlung durch die spezialisierte Palliativmedizin –

Im Folgenden sollen potentielle Situationen beleuchtet werden. Bitte geben Sie an, ob Sie in diesem Fall bei Verfügbarkeit ein Konsil an die spezialisierte Palliativmedizin stellen würden.

**Ich stelle ein Palliativkonsil…**	**Ja**	**Nein**	**k.A.**
wenn mich der Tod des Patienten in den nächsten 12 Monaten nicht überraschen würde („surprise question“)			
wenn die Familie um eine palliativmedizinische Mitbehandlung gebeten hat.			
wenn der Patient um eine palliativmedizinische Mitbehandlung gebeten hat.			
Bei anhaltender, nicht kontrollierbarer Symptomlast des Patienten (z. B. Schmerzen, Dyspnoe)			
wenn der Patient lebensverlängernde oder kurative Therapiemaßnahmen ablehnt.			
wenn der Patient eine mittlere Überlebenswahrscheinlichkeit von weniger als 6 Monaten hat.			
Wenn der Patient bereits an eine SAPV angebunden ist			
Wenn der Patient bei einem früheren Aufenthalt durch den Palliativdienst betreut wurde			
wenn der Patient in der Finalphase ist.			
wenn der Patient einen Todeswunsch äußert oder über Suizidgedanken spricht.			
wenn der Patient einen nicht operablen bösartigen Tumor oder eine Pleura-/Peritonealkarzinose oder eine Meningiosis carcinomatosa hat.			
wenn der Patient die Diagnose eines malignen, hochaktiven Tumors hat.			
wenn dem Patienten die Erstdiagnose eines nicht heilbaren Tumors mitgeteilt wird.			
wenn der Patient über 80 Jahre alt ist und zwei oder mehr lebensbedrohlichen Komorbiditäten vorliegen (z. B. Nierenerkrankung im Endstadium, schweres kongestives Herzversagen).			
wenn der Patient mehrfach im letzten halben Jahr aufgrund großer Symptomlast einer fortschreitenden Erkrankung (z.B. Dyspnoe bei COPD Gold 4 oder Herzinsuffizienz NYHA 4) aufgenommen werden musste			
wenn der Patient an einer progredienten unheilbaren neurologischen Erkrankung leidet (z.B. Chorea Huntington, ALS)			
wenn der Patient freiwillig auf Nahrung und Flüssigkeit verzichtet, um das eigene Leben zu beenden (Sterbefasten).			

Falls kein Palliativkonsil verfügbar ist (Frage 18 mit „nein“ beantwortet): :

Im Folgenden sollen potentielle Situationen beleuchtet werden. Bitte geben Sie an, ob Sie sich in diesen Situationen Unterstützung durch eine/einen SpezialistIn für Palliativmedizin wünschen würden:

**Ich würde mir Unterstützung durch die Palliativmedizin wünschen,**	**Ja**	**Nein**	**k.A.**
wenn mich der Tod des Patienten in den nächsten 12 Monaten nicht überraschen würde („surprise question“)			
wenn die Familie um eine palliativmedizinische Mitbehandlung gebeten hat.			
wenn der Patient um eine palliativmedizinische Mitbehandlung gebeten hat.			
Bei anhaltender, nicht kontrollierbarer Symptomlast des Patienten (z. B. Schmerzen, Dyspnoe)			
wenn der Patient lebensverlängernde oder kurative Therapiemaßnahmen ablehnt.			
wenn der Patient eine mittlere Überlebenswahrscheinlichkeit von weniger als 6 Monaten hat.			
Wenn der Patient bereits an eine SAPV angebunden ist			
wenn der Patient in der Finalphase ist.			
wenn der Patient einen Todeswunsch äußert oder über Suizidgedanken spricht.			
wenn der Patient einen nicht operablen bösartigen Tumor oder eine Pleura-/Peritonealkarzinose oder eine Meningiosis carcinomatosa hat.			
wenn der Patient die Diagnose eines malignen, hochaktiven Tumors hat.			
wenn dem Patienten die Erstdiagnose eines nicht heilbaren Tumors mitgeteilt wird.			
wenn der Patient über 80 Jahre alt ist und zwei oder mehr lebensbedrohlichen Komorbiditäten vorliegen (z. B. Nierenerkrankung im Endstadium, schweres kongestives Herzversagen).			
wenn der Patient mehrfach im letzten halben Jahr aufgrund großer Symptomlast einer fortschreitenden Erkrankung (z.B. Dyspnoe bei COPD Gold 4 oder Herzinsuffizienz NYHA 4) aufgenommen werden musste			
wenn der Patient an einer progredienten unheilbaren neurologischen Erkrankung leidet (z.B. Chorea Huntington, ALS)			
wenn der Patient freiwillig auf Nahrung und Flüssigkeit verzichtet, um das eigene Leben zu beenden (Sterbefasten).			

(Frage 18 mit „ja“ beantwortet“): Einfluss des Palliativmedizinischen (Konsil)-Dienstes

23. Ich fühle mich durch die spezialisierte Palliativmedizin in meiner Arbeit unterstützt.

a) Stimme voll zu
b) Stimme eher zu
c) Neutral
d) Stimme eher nicht zu
e) Stimme gar nicht zu

24. Die Zufriedenheit von Patienten oder Angehörigen wird durch das Hinzuziehen der spezialisierten Palliativmedizin in der Notaufnahme gesteigert.

a) Stimme voll zu
b) Stimme eher zu
c) Neutral
d) Stimme eher nicht zu
e) Stimme gar nicht zu

25. Patienten, die eine Mitbehandlung durch die spezialisierte Palliativmedizin erhalten, haben mittelfristig weniger Beschwerden.

a) Stimme voll zu
b) Stimme eher zu
c) Neutral
d) Stimme eher nicht zu
e) Stimme gar nicht zu

26. Die weiter- oder hauptbehandelnde Abteilung hat häufig Vorbehalte gegen eine Mitbehandlung durch die spezialisierte Palliativmedizin.

a) Stimme voll zu
b) Stimme eher zu
c) Neutral
d) Stimme eher nicht zu
e) Stimme gar nicht zu

27. Wie häufig kommt es vor, dass Patienten oder Angehörige das Hinzuziehen der spezialisierten Palliativmedizin in der Zentralen Notaufnahme ablehnen?

a) Nie
b) Selten
c) Gelegentlich
d) Häufig
e) Immer
f) Keine Angabe/Weiß nicht

Außerklinische Ressourcen

28. Meine Stadt/mein Kreis bietet eine SAPV (spezialisierte ambulante Palliativversorgung) an

a) Ja
b) Nein
c) Weiß ich nicht

29. Ich habe die Möglichkeit, aus der Notaufnahme heraus Patienten in die SAPV einzubinden.

a) Ja
b) Nein
c) Habe ich noch nie versucht
d) Keine Angabe/weiß nicht

30. Falls das SAPV-Team (Ambulantes Palliativteam) Palliativpatienten mit Versorgungsproblemen in das Krankenhaus einweisen will, dann hat es einen festen Ansprechpartner

a) Ja
b) Nein
c) Keine Angabe/Weiß nicht

31. Im Einzugsbereich meiner Notaufnahme gibt es eine standardisierte Verfügung für den Notfall (Notfallausweis/gelber Zettel für den Rettungsdienst/…) mit dem der Rettungsdienst schnell über bestehende Therapielimitierungen informiert werden kann

a) Ja
b) Nein
c) Keine Angabe/Weiß nicht
d) Anderes, nämlich: ________________

32. Die Ermittlung des tatsächlichen Patientenwillens in der Notaufnahme ist zu selten möglich.

a) Stimme voll zu
b) Stimme eher zu
c) Neutral
d) Stimme eher nicht zu
e) Stimme gar nicht zu

Sterbende Patienten in der Zentralen Notaufnahme

33. Patienten (ausgenommen Patienten unter Reanimation oder Polytraume) sollten nicht in der Zentralen Notaufnahme sterben

a) Stimme voll zu
b) Stimme eher zu
c) Neutral
d) Stimme eher nicht zu
e) Stimme gar nicht zu

34. Wie oft sterben Patienten (ausgenommen Patienten unter Reanimation oder Polytrauma) in Ihrer Notaufnahme?

a) Nie
b) Weniger als 1/Monat
c) 1-3/Monat
d) Wöchentlich
e) Mehr als 1/Woche
f) Weiß nicht/keine Angabe

35. Patienten in der Finalphase werden in meiner Notaufnahme auf Station verlegt

a) Immer
b) Häufig
c) Gelegentlich
d) Selten
e) Nie
f) Weiß nicht/keine Angabe

36. Im stressigen Alltag einer Notaufnahme bleibt keine Zeit für die Begleitung von Patienten in der Finalphase.

a) Stimme voll zu
b) Stimme eher zu
c) Neutral
d) Stimme eher nicht zu
e) Stimme gar nicht zu

37. Welche räumlichen Strukturen halten Sie in Ihrer Notaufnahme für sterbende und verstorbene PatientInnen vor? (Mehrfachnennung möglich)

a) Ein eigenes Palliativzimmer für Patienten in der Finalphase und ihre Angehörigen
b) Es gibt Einzelzimmer in der Notaufnahme, die genutzt werden können
c) Es gibt einen Abschiedsraum, in dem sich trauernde Angehörigen von Verstorbenen verabschieden können.
d) Es stehen nur Untersuchungskabinen zur Verfügung
e) In der Notaufnahme gibt es keine abgetrennten geeigneten Räume
f) Sonstiges: ___________________________

38. In belastenden Situationen besteht die Möglichkeit, ein Kriseninterventionsteam oder einen (Notfall-)seelsorger hinzuzuziehen.

a) Ja
b) Nein
c) Keine Angabe/Weiß nicht

39. In meiner Notaufnahme gibt es die Möglichkeit, eine Pflegekraft für die Zeit der Begleitung eines Patienten in der Finalphase freizustellen.

a) Ja
b) Nein
c) Keine Angabe/Weiß nicht

40. In meiner Notaufnahme gibt es ein Training für die Gesprächsführung in schwierigen medizinischen Situationen.

a) Ja
b) Nein
c) Keine Angabe/Weiß nicht

41. In meiner Notaufnahme gibt es eine regelmäßige Supervision für KollegInnen nach belastenden Situationen.

a) Ja
b) Nein
c) Keine Angabe/Weiß nicht
